# A Novel Xenomonitoring Technique Using Mosquito Excreta/Feces for the Detection of Filarial Parasites and Malaria

**DOI:** 10.1371/journal.pntd.0004641

**Published:** 2016-04-20

**Authors:** Nils Pilotte, Weam I. Zaky, Brian P. Abrams, Dave D. Chadee, Steven A. Williams

**Affiliations:** 1 Department of Biological Sciences, Smith College, Northampton, Massachusetts, United States of America; 2 Molecular and Cellular Biology Program, University of Massachusetts Amherst, Amherst, Massachusetts, United States of America; 3 Department of Science, Groton School, Groton, Massachusetts, United States of America; 4 Department of Life Sciences, University of the West Indies, St Augustine, Trinidad and Tobago, West Indies; Vienna, AUSTRIA

## Abstract

**Background:**

Given the continued successes of the world’s lymphatic filariasis (LF) elimination programs and the growing successes of many malaria elimination efforts, the necessity of low cost tools and methodologies applicable to long-term disease surveillance is greater than ever before. As many countries reach the end of their LF mass drug administration programs and a growing number of countries realize unprecedented successes in their malaria intervention efforts, the need for practical molecular xenomonitoring (MX), capable of providing surveillance for disease recrudescence in settings of decreased parasite prevalence is increasingly clear. Current protocols, however, require testing of mosquitoes in pools of 25 or fewer, making high-throughput examination a challenge. The new method we present here screens the excreta/feces from hundreds of mosquitoes per pool and provides proof-of-concept for a practical alternative to traditional methodologies resulting in significant cost and labor savings.

**Methodology/Principal Findings:**

Excreta/feces of laboratory reared *Aedes aegypti* or *Anopheles stephensi* mosquitoes provided with a *Brugia malayi* microfilaria-positive or *Plasmodium vivax*-positive blood meal respectively were tested for the presence of parasite DNA using real-time PCR. A titration of samples containing various volumes of *B*. *malayi*-negative mosquito feces mixed with positive excreta/feces was also tested to determine sensitivity of detection. Real-time PCR amplification of *B*. *malayi* and *P*. *vivax* DNA from the excreta/feces of infected mosquitoes was demonstrated, and *B*. *malayi* DNA in excreta/feces from one to two mf-positive blood meal-receiving mosquitoes was detected when pooled with volumes of feces from as many as 500 uninfected mosquitoes.

**Conclusions/Significance:**

While the operationalizing of excreta/feces testing may require the development of new strategies for sample collection, the high-throughput nature of this new methodology has the potential to greatly reduce MX costs. This will prove particularly useful in post-transmission-interruption settings, where this inexpensive approach to long-term surveillance will help to stretch the budgets of LF and malaria elimination programs. Furthermore, as this methodology is adaptable to the detection of both single celled (*P*. *vivax*) and multicellular eukaryotic pathogens (*B*. *malayi*), exploration of its use for the detection of various other mosquito-borne diseases including viruses should be considered. Additionally, integration strategies utilizing excreta/feces testing for the simultaneous surveillance of multiple diseases should be explored.

## Author Summary

As a non-invasive method of indirectly monitoring insect-borne disease, molecular xenomonitoring (MX), the molecular testing of insects for the presence of a pathogen, can provide important information about disease prevalence without the need for human sampling. However, given the successes of tropical disease elimination programs, including many lymphatic filariasis and malaria elimination efforts, parasite levels in many locations are declining. This decrease in prevalence requires the sampling of increased numbers of vectors for disease surveillance and recrudescence monitoring. Such increased sampling poses a challenge since it results in additional costs and labor. In light of these difficulties, high-throughput methodologies for MX are necessary to provide elimination programs with cost-reducing alternatives to long-term disease surveillance. Here we demonstrate proof-of-concept for a new method that samples large numbers of mosquitoes using PCR to screen excreta/feces for filarial or malarial parasites. If operationalized, this approach to MX will provide a practical “first-alert” system that will enable cost-minimizing surveillance in post-transmission-interruption settings. Given this potential, the applicability of this approach to the monitoring of various mosquito-borne diseases should be explored further, as this platform will prove useful for surveillance efforts for a wide variety of pathogens.

## Introduction

Spanning 73 countries and territories and placing an estimated 1.39 billion individuals at risk of infection, lymphatic filariasis (LF) presents a considerable risk to global health [1]. Similarly, with an estimated 198 million malaria infections and 584,000 malaria-related deaths in 2013, the global burden of human malaria is staggering [2]. Yet despite the wide ranging impacts of these diseases, global elimination efforts have made significant strides, spearheaded by mass drug administration (MDA) programs supported by large pharmaceutical donors [3–5] and the widespread use of insecticidal bed nets [6–9]. As a result, disease prevalence in many locations has decreased dramatically, enabling a growing number of countries to discontinue their treatment efforts for LF [5, 10] and spurring the creation of an increasing number of malaria elimination programs [11–13]. However, lessons learned as a result of LF elimination efforts have shown that the cessation of MDA, recommended after the successful passing of a transmission assessment survey [14], results in an additional set of programmatic challenges. Foremost in such post-intervention settings is the issue of post-MDA surveillance, as vigilant monitoring is required to ensure that recrudescence of disease has not occurred [15]. This monitoring is costly and current efforts for LF are centered upon the periodic sampling of the human population in order to examine circulating levels of filarial antigen [16–17]. While effective, these efforts require blood sampling of the human population. The invasive nature of this practice, coupled with the requirement of informed consent, results in participation challenges [14] that logically increase as populations become further removed from the time of widespread disease transmission. While still largely of future concern, similar challenges likely await the malaria community as control efforts continue to reduce the burden of disease, making this programmatic obstacle one of utmost global importance.

Molecular xenomonitoring (MX), the testing of vectors for the presence of parasite genetic material, has been proposed as a non-invasive means of conducting post-MDA surveillance for LF [14, 17–18]. Although precise correlations between levels of parasite within the vector population and levels within the human population have not been conclusively established, parasite presence within the vector population is indicative of the potential for disease transmission. Furthermore, when monitoring for LF in locations endemic for the *Wuchereria bancrofti* parasite, a pathogen without a known zoonotic host [19], presence is directly indicative of active human infection. Yet despite its many advantages, MX is costly and when used for monitoring in a post-MDA setting, typically requires the collection and sampling of many thousands of mosquitoes [18, 20–21]. Therefore, as a growing number of countries continue to enter the surveillance phases of their LF eradication programs, alternative methodologies for streamlining, simplifying, and reducing the costs associated with post-MDA monitoring will be required.

As an alternative to traditional approaches to MX, excreta and feces produced by mosquitoes potentially harboring parasites can be tested for the presence of pathogen DNA. Previous work has demonstrated that vector feces-monitoring for the PCR-based detection of *Trypanosoma cruzi* can be used as a means of surveying insect host infection status [22]. Similarly, it has been shown that genetic material from the *Brugia malayi* parasite can be successfully detected in the excreta and feces collected from individual mosquitoes [23]. Building upon these findings, we describe methodological proof-of-principle for the real-time PCR-based monitoring for *B*. *malayi* parasite DNA in pools of mosquito excreta/feces as a platform for the surveillance of large numbers of insects. While unconventional, excreta/feces monitoring has the potential to provide significant time, cost, and labor savings over traditional MX methodologies due to its exceptionally high-throughput nature. Furthermore, as excreta/feces collection would likely prove readily adaptable to a variety of both passive and active trapping practices and platforms, its potential feasibility as an exceedingly low cost, long-term surveillance tool is great. Equally promising, initial experiments have demonstrated that this approach to MX can be applied to the detection of *Plasmodium vivax* DNA, indicating its possible usefulness for the monitoring of both unicellular and multicellular eukaryotic pathogens. Given these encouraging findings, the further exploration of mosquito excreta/feces testing as a new method for disease surveillance purposes is warranted and efforts to adapt this alternative MX approach to other mosquito-borne illnesses should be pursued.

## Materials and Methods

### Mosquito Rearing for the Accumulation of Excreta/Feces

#### Accumulation of excreta/feces from mosquitoes potentially infected with *B*. *malayi*

Mosquito cartons containing the excreta/feces from female *Aedes aegypti* mosquitoes potentially infected with the *B*. *malayi* parasite were received from the Filarial Research Reagent Resource Center (FR3) located at the University of Georgia, College of Veterinary Medicine, Athens, GA. Rearing and infection of mosquitoes occurred in accordance with “SOP Number 8.3” available on the FR3 website (http://www.filariasiscenter.org/parasites-resources/Protocols/materials-1).

#### Accumulation of excreta/feces from mosquitoes potentially infected with *P*. *vivax*

Mosquito cartons containing the excreta/feces from female *Anopheles stephensi* mosquitoes potentially infected with the *P*. *vivax* parasite were received from the Centers for Disease Control and Prevention, Atlanta, GA, USA. Rearing of mosquitoes occurred in accordance with the protocols described in chapter 2.4 of the “Methods in *Anopheles* Research” manual [24] available on the BEI Resources website (https://www.beiresources.org/Catalog/VectorResources.aspx). Infection of mosquitoes occurred in accordance with previously described methodologies [25].

#### Accumulation of feces from uninfected mosquitoes

Moist filter paper rafts containing *Culex quinquefasciatus* eggs were received from BEI Resources (www.beiresources.org). Eggs were rinsed into open-topped plastic vessels containing approximately 1 L of tap water at a depth of approximately 5 cm and a small volume of standard flake-based fish food was added to the water in each container. Upon maturation into pupae, 50 mosquitoes were transferred into plastic containers, approximately 5 cm in diameter, containing 1 cm of tap water. These containers were then placed into waxed cardboard cartons (approximately 18 cm in diameter by 14.5 cm in height). Cartons were covered with standard mesh tulle and mosquitoes were allowed to emerge as adults. Upon emergence, a cotton ball soaked in 10% sucrose was placed on top of each carton and this solution was refreshed daily. Mosquitoes remained within the cartons producing feces until they expired naturally (10–20 days). At this time, the expired mosquitoes were removed and the cartons were collected, flattened, and stored at 4°C.

### Extraction of DNA from Mosquito Excreta/Feces

Preliminary experiments were designed to determine the effectiveness/efficiency of extracting DNA from the excreta/feces of mosquitoes potentially infected with the *B*. *malayi* parasite. To make this determination, various extraction protocols and techniques were tested in order to evaluate their efficiency (Table 1). Because the FR3-derived mosquito cartons containing excreta/feces from potentially infected insects were non-waxed, initial samples were either scraped off of the cartons using a metal spatula, or strips of the carton material (hereafter referred to as carton strips) were directly used as the starting material for the extraction procedure. The amplification of *B*. *malayi* parasite DNA from all extracts was evaluated using the previously described real-time PCR primer-probe pairing [26]. Results demonstrated that DNA extractions performed using the QIAamp DNA Micro Kit (Qiagen, Valencia, CA) provided the most consistent and effective detection of parasite DNA. For this reason, this kit was used in all subsequent experiments.

**Table 1:** Evaluation of extraction methods for the isolation of DNA from mosquito excreta/feces.

DNA Extraction Method	Quantity of Excreta/Feces (Mosquito Excreta/Feces/Days)*	# of Samples (# of Positives)
Qiagen DNeasy Blood and Tissue (Qiagen, Valencia, CA)	62.5	2 (0)
Overnight Soak in 1 x PBS	62.5	8 (0)
Overnight Soak in 1 x TE	62.5	8 (0)
Phire Plant Direct PCR Kit (Thermo Fisher Scientific, Vantaa, Finland)	62.5	8 (0)
Published Insect Feces Extraction^[^^22^^]^	1–2	5 (0)
Published Insect Feces Extraction^[^^22^^]^ + Phenol/Chisam Purification	1–2	5 (0)
QIAamp DNA Micro Kit Extraction (Qiagen, Valencia, CA)	1–2	5 (5)
Nucleospin Blood DNA Kit (Macherey-Nagel, Bethlehem, PA)	1–2	5 (0)
Nucleospin Blood DNA Kit (Macherey-Nagel, Bethlehem, PA)	1–2	5 (0)

* Mosquito Excreta/Feces/Days are defined as the estimated quantity of excreta/feces produced by a single mosquito over a 24 hour period.

To adapt the Qiagen protocol for use with the bulky, brittle mosquito carton material, minor modifications were made to the manufacturer’s suggested instructions for DNA extraction from bloodspots. Briefly, carton strips were soaked in 360 μl of Buffer ATL for 1 hour prior to incubation with Proteinase K at 56°C. Additionally, following incubation at 70°C, samples were centrifuged at maximum speed for 5 min and supernatants were transferred to new 1.7 ml microcentrifuge tubes. Tubes were centrifuged for an additional 5 min at maximum speed to pellet residual debris and the supernatants were transferred to QIAamp MinElute columns. Lastly, all samples were incubated in Buffer AE at room temperature for 5 min prior to the elution of samples from the columns.

### Evaluation of Positivity of Excreta/Feces from Mosquitoes Potentially Infected with *B*. *malayi*

Although preliminary experiments demonstrated that excreta/feces derived from vector mosquitoes fed on *B*. *malayi* microfilaria (mf)-positive blood resulted in the amplification of parasite DNA, the availability of mf-containing blood does not guarantee that all mosquitoes will feed or ingest parasites while feeding. Additionally, as the FR3’s standard operating procedure (SOP 8.3) requires that mosquitoes spend three to five days as adults prior to the time an infective blood meal is introduced, a substantial volume of parasite-negative feces was produced and deposited into mosquito cartons prior to blood feeding. Furthermore, as mosquitoes are known to excrete while taking a blood meal [27], it is likely that excreta would be deposited before parasite DNA had reached/been incorporated into the voided material. Therefore, a portion of the voided material collected from mosquitoes provided with mf-positive blood would likely not contain parasites and would therefore not result in a positive PCR. For this reason, a large panel of potentially positive excreta/feces samples was tested in order to estimate the rates of sample positivity. In total, 59 independent samples were tested, with each sample consisting of a 0.48 cm^2^ carton strip. Based upon observations of the volume of excreta/feces produced by single mosquitoes housed in 50 ml conical tubes, it was estimated that the volume of excreta/feces on each carton strip was equivalent to the average volume produced by one to two mosquitoes over a 24 hour period. Negative control extractions were performed on similar volumes of mosquito feces collected from uninfected *C*. *quinquefasciatus*. All samples underwent DNA extraction using the modified Qiagen procedure described above and were analyzed by 45 cycles of real-time PCR using the published reagent concentrations and cycling protocol [26]. 2 μl aliquots of each DNA extract were tested in triplicate and samples returning two or more positive results were considered positive for *B*. *malayi* parasite DNA.

### Assay Sensitivity Testing

In order to determine detection limits for the presence of *B*. *malayi*-infected excreta/feces in large pools of uninfected mosquito feces, a titration of samples was created, with each sample containing a 0.48 cm^2^ strip from a carton used to house mosquitoes provided with a *B*. *malayi-*positive blood meal mixed with various volumes of uninfected mosquito feces. Feces from uninfected *C*. *quinquefasciatus* mosquitoes were removed from cartons using a cotton swab, and the feces-covered cotton was added to each sample. As 50 uninfected mosquitoes were raised in each carton, and adult mosquitoes were observed to survive for a minimum of 10 days (with most surviving considerably longer), it was conservatively estimated that each carton contained a minimum of 500 mosquito feces/days (i.e. the amount of feces produced by 500 mosquitoes in one 24 hour period, or the amount of feces produced by a single mosquito over a 500 day period). While the distribution of feces within cartons was not precisely uniform, by sectioning cartons based upon total internal surface area (approximately 1,050 cm^2^), it was possible to roughly estimate the number of mosquito feces/days being added to each sample. Samples estimated to contain approximately 62.5, 125, 250, and 500 feces/days were prepared. Negative control extractions were also prepared using mosquito feces collected from uninfected *C*. *quinquefasciatus*. All samples were extracted and tested in duplicate reactions using the same extraction and detection methods as described above for the evaluation of PCR positivity testing.

### Adaptation of Excreta/Feces Testing to the Detection of *P*. *vivax* DNA

To test whether the detection of mosquito-borne pathogen DNA from mosquito excreta/feces was possible for species other than the *B*. *malayi* parasite, a set of samples was created using mosquito excreta/feces produced by carton-raised *A*. *stephensi* that had been fed on *P*. *vivax*-positive blood. As was done for *B*. *malayi* detection, samples were prepared by excising 0.48 cm^2^ carton strips containing potentially positive excreta/feces. To establish proof-of-principle, 20 samples were prepared and DNA was extracted using the modified Qiagen protocol described above. DNA extracts from each sample were tested using a previously described primer-probe set for the universal detection of *Plasmodium* species [28] with reaction recipes and cycling conditions remaining consistent with the authors’ published protocol.

## Results

### Evaluation of PCR Positivity of Excreta/Feces from Mosquitoes Potentially Infected with *B*. *malayi*

Carton strips were excised from containers used to house *A*. *aegypti* mosquitoes provided with *B*. *malayi* mf-containing blood and testing was conducted to determine the percentage of excreta/feces samples containing *B*. *malayi* DNA. Such testing was necessary since the production of feces can occur prior to the provision of an infective blood meal or before the ingestion of a blood meal. Furthermore, the availability of infective blood does not guarantee that each individual mosquito will feed and, dependent upon the mosquito species, localization of parasite material to voided excreta/feces may take time following blood meal ingestion. Accordingly, DNA was extracted from 59 independent samples, each consisting of a carton strip measuring 0.48 cm^2^ and containing excreta/feces from one to two mosquitoes over a 24 hour period (i.e. one to two mosquito feces/days). Real-time PCR testing, using 2 μl of template DNA resulted in positive detection for 21 out of 59 samples tested (35.6%). For positive samples, mean Ct values ranged from 26.62 (+/- 0.24) to 41.98 (+/- 0.03) (Table 2). Because only a fraction of the deposited mosquito excreta/feces would contain parasite DNA, 35.6% may be a true indication of the frequency of positive samples.

**Table 2:** PCR positivity of excreta/feces from mosquitoes potentially infected with *B*. *malayi*.

Sample #	Ct Value (Std. Dev.)	Sample #	Ct Value (Std. Dev.)
1	Negative	32	Negative
2	27.75 (+/- 0.15)	33	Negative
3	28.67 (+/- 0.17)	34	Negative
4	Negative	35	Negative
5	38.25 (+/- 2.79)	36	Negative
6	Negative	37	Negative
7	Negative	38	Negative
8	36.53 (+/- 2.33)	39	39.07 (+/- 1.07)
9	40.49 (+/- 0.12)	40	Negative
10	Negative	41	Negative
11	34.80 (+/- 0.70)	42	Negative
12	37.55 (+/- 0.91)	43	Negative
13	41.96 (+/- 2.67)	44	40.74 (+/- 2.17)
14	40.54 (+/- 1.26)	45	Negative
15	Negative	46	31.69 (+/- 0.50)
16	Negative	47	Negative
17	Negative	48	38.88 (+/- 0.49)
18	Negative	49	37.69 (+/- 0.69)
19	Negative	50	Negative
20	Negative	51	40.32 (+/- 0.43)
21	Negative	52	Negative
22	Negative	53	37.49 (+/- 1.53)
23	Negative	54	Negative
24	Negative	55	39.48 (+/- 2.37)
25	41.44 (+/- 0.95)	56	26.62 (+/- 0.24)
26	27.91 (+/- 0.54)	57	Negative
27	Negative	58	Negative
28	41.98 (+/- 0.03)	59	Negative
29	Negative	Negative Extract #1	Negative
30	Negative	Negative Extract #2	Negative
31	Negative		

### Assay Sensitivity Testing

A titration of samples containing potentially positive 0.48 cm^2^ carton strips mixed with varying amounts of uninfected mosquito feces was prepared in order to estimate the limits of detection for *B*. malayi-based excreta/feces testing. In total, five samples containing an estimated 62.5 mosquito feces/days, six samples containing an estimated 125 mosquito feces/days, six samples containing an estimated 250 mosquito feces/days, and two samples containing an estimated 500 mosquito feces/days were assayed. As expected, due to the uncertainty of which samples actually contained *B*. *malayi* DNA, a fraction of the samples failed to give positive PCR detection of *B*. *malayi* DNA. However, detection of parasite DNA proved possible at all tested levels of sensitivity (Table 3).

**Table 3:** Limits for the detection of *B*. *malayi* DNA in mosquito excreta/feces samples.

Sample ID	Quantity of Potentially Positive Excreta/Feces (Mosquito Excreta/Feces/Days)*	Quantity of Negative Excreta/Feces (Mosquito Excreta/Feces/Days)*	Ct Value (Std. Dev.)
A	1–2	62.5	Negative
B	1–2	62.5	Negative
C	1–2	62.5	Negative
D	1–2	62.5	37.89 (+/- 2.31)
E	1–2	62.5	38.77 (+/- 0.20)
F	1–2	125	30.98 (+/- 0.20)
G	1–2	125	Negative
H	1–2	125	Negative
I	1–2	125	Negative
J	1–2	125	Negative
K	1–2	125	Negative
L	1–2	250	29.56 (+/- 0.01)
M	1–2	250	35.88 (+/- 0.04)
N	1–2	250	Negative
O	1–2	250	38.73 (+/- 0.91)
P	1–2	250	Negative
Q	1–2	250	Negative
R	1–2	500	Negative
S	1–2	500	38.40 (+/- 1.03)
Negative #1	N/A	62.5	Negative
Negative #2	N/A	62.5	Negative
Negative #3	N/A	62.5	Negative

* Mosquito Excreta/Feces/Days are defined as the estimated quantity of excreta/feces produced by a single mosquito over a 24 hour period.

### Adaptation of Excreta/Feces Testing to the Detection of *Plasmodium vivax* DNA

To explore whether excreta/feces testing would efficiently detect pathogen DNA from species other than *B*. *malayi*, testing for the presence of the human malaria-causing parasite *P*. *vivax* was performed. To demonstrate proof-of-concept, 20 samples were prepared and tested by PCR. Each sample was comprised of a 0.48 cm^2^ carton strip excised from a mosquito container having housed *A*. *stephensi* female mosquitoes provided with *Plasmodium*-positive blood. Real-time PCR testing of DNA extracted from each sample clearly demonstrated the adaptability of excreta/feces testing to the detection of *P*. *vivax* since all samples were positive with Ct values ranging from 26.82 (+/- 0.26) to 29.21 (+/- 0.80) (Table 4).

**Table 4:** PCR positivity of excreta/feces from mosquitoes infected with *P*. *vivax*.

Sample #	Ct Value (std. dev.)	Sample #	Ct Value (std. dev.)
1	28.23 (+/- 0.58)	12	28.77 (+/- 0.09)
2	27.84 (+/- 0.05)	13	28.83 (+/- 0.49)
3	27.85 (+/- 0.15)	14	27.88 (+/- 0.19)
4	27.85 (+/- 0.29)	15	27.36 (+/- 0.14)
5	26.82 (+/- 0.26)	16	26.82 (+/- 0.10)
6	27.98 (+/- 0.28)	17	28.00 (+/- 0.27)
7	26.94 (+/- 0.20)	18	28.48 (+/- 0.29)
8	27.26 (+/- 0.32)	19	28.40 (+/- 0.26)
9	27.19 (+/- 0.32)	20	28.07 (+/- 0.12)
10	28.37 (+/- 0.49)	Negative Extract #1	Negative
11	29.21 (+/- 0.80)	Negative Extract #2	Negative

## Discussion

While sensitive and less intrusive to the local population than human sampling, the number of studies implementing current MX practices for the surveillance of LF or malaria has been limited. Although such efforts provide valuable data [10, 17–18, 21] the routine use of MX for post-MDA LF surveillance or long-term recrudescence monitoring is not yet standard procedure. Despite the existence of effective molecular tools [28–29], vector monitoring for malaria is even more uncommon and World Health Organization recommendations for infection monitoring and prevalence estimation rely solely on human sampling [2]. Limited implementation has occurred for multiple reasons, including the need to process and test large numbers of mosquitoes from areas suspected of having low parasite density within the vector population [10, 18, 21]. Difficulties in establishing a concrete correlation between vector-parasite levels and human prevalence have further restricted MX implementation [21]. Yet despite these shortcomings, MX continues to receive attention as the need for post-intervention disease surveillance continues to grow and mosquito trap designs continue to improve [30–34]. Accordingly, methodologies capable of harnessing the advantageous aspects of MX while making its practice more practical and inexpensive would be of great benefit to global LF and malaria elimination efforts, as well as to monitoring efforts for other vector-borne diseases.

The work presented here provides methodological proof-of-concept for a novel approach to MX with the potential to greatly reduce the cost, time, and labor associated with large-scale surveillance efforts. The successful amplification of parasite DNA from pooled mosquito excreta/feces containing *B*. *malayi* genetic material has demonstrated that high-throughput MX for LF is feasible. In the past, real-time PCR-based MX for the presence of the filariasis-causing parasites has been restricted to the testing of pools of 25 or fewer mosquitoes. This is because the biological mass of mosquitoes and high yields of mosquito DNA associated with pools of large size results in the inability to detect the presence of small quantities of parasite DNA [35]. However, excreta/feces testing enables the sampling of material obtained from vast numbers of mosquitoes, while simultaneously limiting the biological mass associated with each sample. As we have demonstrated, it is possible to detect trace amounts of parasite DNA in pools containing the voided material from as many as 500 uninfected mosquitoes. Future studies implementing this approach will benefit from the drastic reduction in cost of DNA extractions and PCR (approximately 20-fold). Furthermore, as it has been shown that non-vector mosquitoes rid themselves of parasite material more rapidly than vector species (as indicated by a shortened period of time during which parasite detection is possible within non-vectors [23]), one would expect to find greater quantities of parasite DNA within the excreta/feces of non-vector mosquitoes. Therefore, the testing of mixed pools of vector and non-vector excreta/feces should be possible. While such testing will result in reduced ability to directly correlate the presence of parasite with individual vector species, it will likely increase the sensitivity of detection when surveying for the presence of parasite in post-transmission-interruption settings as both vector and non-vector mosquitoes potentially harboring parasite material will be screened. In addition, it is likely that excreta/feces testing will eliminate the need for the labor intensive and time consuming species-sorting efforts which are commonplace in current MX work [10, 17–18, 21, 36]. By drastically reducing the numbers of pools that must be screened and by eliminating the need for sorting mosquitoes by species, labor-related time and costs are dramatically reduced.

While operationalizing this alternative approach to MX presents some implementation hurdles, adaptation of current passive and active trapping methods to the collection of mosquito excreta/feces is possible. Such adaptation could occur by transferring live mosquitoes from a trap to a holding carton, in which they would be sugar fed using a cotton ball, thereby encouraging the voiding of waste material. Expired mosquitoes would then be removed and additional mosquitoes could be added following further collection from the trap. Periodic testing of the accumulated excreta/feces would enable the high-throughput screening of the voided material from a series of such traps. Any trap with the capacity to maintain live mosquitoes could be used for this purpose including the CDC Gravid Trap, the Ifakara tent trap and others [30, 37]. Alternatively, collection of excreta/feces could occur directly within traps of various designs. One such design proving readily adaptable to excreta/feces collection in preliminary experiments is the “Large Passive Box Trap” developed by Ritchie, et al [38]. While work aimed at evaluating the adaptability of this trap to the collection of various species of mosquitoes is currently ongoing, and further efforts to optimize this trap for the purpose of excreta/feces collection will be required, simply lining the internal surfaces of this passive trap with waxed paper provides an uncomplicated method for collecting the accumulated material voided by trapped mosquitoes (S1 Fig). Swabbing the excreta/feces from the waxed paper then enables the PCR analysis of pooled material.

Additional testing will be required to determine the stability of parasite DNA in mosquito excreta/feces over time and under field conditions. However, in the proof-of-concept experiments described in this paper, mosquito excreta/feces containing parasite DNA was allowed to accumulate for 14–16 days prior to transfer to cold storage. In this setting, parasite DNA remained stable and detectable (Table 3). While further validation under conditions mimicking tropical temperatures and humidity will be required, these results are encouraging, as DNA stability within tropical and sub-tropical climates could present another hurdle when operationalizing this method in the field.

Since production of feces can occur prior to the provision of a parasite-positive blood meal and since this provision does not ensure that all mosquitoes will ingest and/or metabolize a parasite, a percentage of the excreta/feces samples collected will likely test negative for parasite DNA. It is therefore difficult using blood-fed mosquitoes to definitively assess the consistency of detection of parasite DNA in excreta/feces. During initial testing, we demonstrated that 21 out of 59 samples comprised of 0.48 cm^2^ carton strips derived from containers used to rear mosquitoes with a *B*. *malayi*-positive blood source were positive (Table 2). However, although sufficient to fulfill our primary aim of providing methodological proof-of-concept, it cannot be conclusively determined whether the remaining 38 samples were all truly negative for parasite DNA. While spiking uninfected excreta/feces samples with extracted *B*. *malayi* genomic DNA would provide clear positive and negative samples, this approach is extremely artificial and has decreased biological relevance since it eliminates any possible effects of mosquito metabolism on the integrity of parasite DNA. Since the major uses of excreta/feces testing will likely center on mapping and long-term, low-cost, post-transmission-interruption recrudescence monitoring, marginally reduced consistency of detection has diminished significance as continuous, sustainable, high-throughput surveillance would enable detection of even low-levels of parasite prevalence. The high-throughput nature of this testing was clearly demonstrated by the positive detection of parasite DNA derived from pools containing various volumes of negative feces up to 500 mosquito feces/days (Table 3). Detection proved possible at all tested sensitivity levels and with overall sample positivity rates similar to those obtained when testing potentially positive excreta/feces samples without the addition of negative feces (36.8% vs. 35.6% respectively). Thus, the inclusion of large amounts of negative feces does not appear to alter detection efficiency. Given these findings, sustainable, high-throughput surveillance efforts using excreta/feces screening could serve as a “first-alert” platform, with positive detection serving as a “red flag” for recrudescence in settings of known transmission interruption. In such a scenario, detection would spur the implementation of more traditional surveillance and monitoring studies.

By successfully detecting *P*. *vivax* DNA in pools of excreta/feces produced by *Plasmodium*-positive-blood fed *A*. *stephensi*, we have provided proof-of-principle for the application of this platform to the detection of malaria parasites. Furthermore, the increased rates of sample positivity and decreased Ct values seen when assaying for *P*. *vivax* are not entirely surprising and indicate this system may work even better for malaria than LF. Estimates have suggested that the ratio of *Plasmodium* merozoites to gametocytes within the peripheral blood is as great as 156:1 [39–40]. Given this ratio, the vast number *Plasmodium* merozoites ingested during a blood meal (up to 32 per infected erythrocyte [41]), and knowledge that merozoites obtained during blood feeding are unable to undergo further development within the mosquito host (only gametocytes undergo further development [42]), the great majority of ingested parasites are simply metabolized and/or eliminated by the mosquito. In contrast, while mosquito hosts possess measures that provide partial protection against filarial infection [43–44], and environmental conditions are thought to impact rates of parasite survival [45], all filarial parasites taken up as part of a blood meal are of the correct lifecycle stage (mf) to potentially undergo further development within the vector host. Therefore, due to the varying natures of their lifecycles, it follows that a greater percentage of filarids ingested during a blood meal are able to successfully develop within the mosquito host as compared to *Plasmodium*. Since successful parasite development would likely mean the absence of parasite DNA in mosquito excreta/feces, the lower levels of sample positivity and the more modest Ct values observed during *B*. *malayi* testing compared to *P*. *vivax* testing seem logical.

With its adaptability to both *B*. *malayi* and *P*. *vivax*, MX of mosquito excreta/feces for various other mosquito-borne pathogens should be explored. Given the successes realized with the detection of these parasites, it is extremely likely that similar detection will prove possible for *W*. *bancrofti* and other malaria species. However, the applicability of this new platform to other types of pathogens should also be examined, since improved high-throughput screening for RNA viruses such as Dengue, Chikungunya, and Zika would be welcomed programmatic tools. Furthermore, since all species of biting insects draw from the same reservoir of blood within a target host, the possibility of cross-vector monitoring should also be considered. For example, excreta/feces samples collected from mosquitoes could be monitored for the presence of disease-causing agents having unrelated insect hosts (such as *Leishmania* ssp. or *Loa loa*). Adaptability to various pathogens and the possibility of cross-vector monitoring could also make excreta/feces sampling an attractive strategy for tropical disease integration efforts. In light of these factors, and the potential time, cost, and labor savings associated with such applications, we believe that this proof-of-concept study suggests that further evaluation of this new method is warranted.

## Supporting Information

S1 Fig Mosquito excreta/feces collection in the modified “Large Passive Box Trap”. Voided material from mosquitoes entering the passive trap collects on wax paper lining the trap surfaces below. Outlined in red is one such mosquito and a series of excreta/feces “spots” which has accumulated.
(TIF)
