# Supplementary figures and images for: A Novel Xenomonitoring Technique Using Mosquito Excreta/Feces for the Detection of Filarial Parasites and Malaria
Source: PLoS Negl Trop Dis. 2016 Apr 20;10(4):e0004641. doi: 10.1371/journal.pntd.0004641 (PMC4838226; doi:10.1371/journal.pntd.0004641)

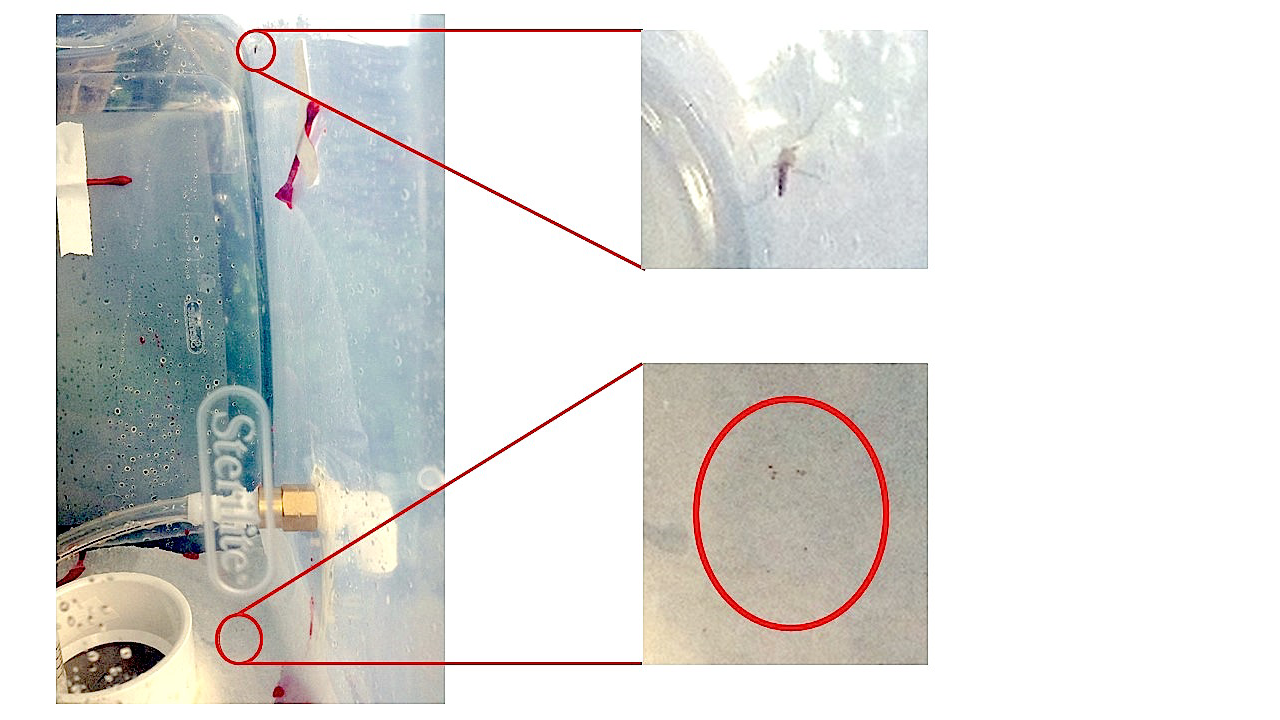

Supplement: S1 Fig — Voided material from mosquitoes entering the passive trap collects on wax paper lining the trap surfaces below. Outlined in red is one such mosquito and a series of excreta/feces “spots” which has accumulated. (TIF) [file pntd.0004641.s001.tif]
